# DeepDeconUQ estimates malignant cell fraction prediction intervals in bulk RNA-seq tissue

**DOI:** 10.1371/journal.pcbi.1013133

**Published:** 2025-06-04

**Authors:** Jiawei Huang, Yuxuan Du, Kevin R. Kelly, Jinchi Lv, Yingying Fan, Jiang F. Zhong, Fengzhu Sun

**Affiliations:** 1 Department of Quantitative and Computational Biology, University of Southern California, Los Angeles, California, United States of America; 2 Department of Electrical and Computer Engineering, University of Texas at San Antonio, San Antonio, Texas, United States of America; 3 Division of Hematology, University of Southern California, Los Angeles, California, United States of America; 4 Data Sciences and Operations Department, University of Southern California, Los Angeles, California, United States of America; 5 Department of Basic Sciences, School of Medicine, Loma Linda University, Loma Linda, California, United States of America; Tsinghua University, CHINA

## Abstract

Accurate estimation of malignant cell fractions in tissues plays a critical role in cancer diagnosis, prognosis, and subsequent treatment decisions. However, most currently available methods provide only point estimates, neglecting the quantification of uncertainties, which is essential for both clinical and research applications. This study introduces DeepDeconUQ, a deep neural network model developed to estimate prediction intervals for malignant cell fractions based on bulk RNA-seq data. This approach addresses limitations in current malignant cell fraction estimation methods by integrating uncertainty quantification into predictions of cancer cell fractions. DeepDeconUQ leverages single-cell RNA sequencing (scRNA-seq) data in conjunction with conformalized quantile regression to produce reliable prediction intervals. The model trains a quantile regression neural network to establish upper and lower bounds for cancer cell proportions, followed by a calibration step that refines these intervals to ensure both statistical validity (coverage probability) and discrimination (narrow intervals). Benchmark analyses indicate that DeepDeconUQ consistently surpasses existing methods, achieving high coverage accuracy with tight prediction intervals across simulated and real cancer datasets. The robustness of DeepDeconUQ is further demonstrated by its resilience to various gene expression perturbations. The DeepDeconUQ method is publicly accessible at https://github.com/jiaweih14/DeepDeconUQ.

## Author summary

Accurately determining the proportion of malignant cells in tumor tissues is crucial for cancer diagnosis and treatment planning. Current methods often provide single estimates without indicating the uncertainty, which can lead to overconfidence in clinical decisions. Here, we present DeepDeconUQ, a deep learning tool that not only predicts the fraction of malignant cells in bulk RNA sequencing data but also quantifies the uncertainty around these estimates. By leveraging single-cell RNA sequencing data to simulate realistic tumor samples, DeepDeconUQ trains a neural network to generate prediction intervals—ranges within which the true malignant cell fraction is likely to lie with high probability. This approach combines quantile regression and statistical calibration to ensure reliability without restrictive assumptions about data distribution. When tested on both simulated and real-world datasets, DeepDeconUQ consistently outperformed existing methods, delivering precise intervals that reliably capture true values while remaining robust against technical noise in gene expression measurements. Our tool addresses a critical gap in cancer genomics by providing clinicians and researchers with confidence intervals that enhance the interpretability of bulk tissue analyses. This advancement could improve personalized treatment strategies and reduce errors in downstream research applications.

## Introduction

Recent advancements in next-generation sequencing methodologies, particularly bulk RNA sequencing (RNA-seq) and single-cell RNA sequencing (scRNA-seq), have substantially driven progress across biological and medical research domains [1–4]. One prominent application is to estimate malignant cell fraction from bulk RNA-seq samples [5–9]. This process typically involves using regression-based methods that leverage malignant and normal expression data (e.g., scRNA-seq) as a reference profile [10]. Most available estimation methods merely provide point estimates of cell-type proportions from bulk RNA-seq data [5, 6]. The accuracy of these methods often depends on the choice and quality of the reference profile [8]. Furthermore, limited efforts have been made to investigate and quantify the impacts of uncertainties in estimated cell-type proportions, which can critically impact downstream analyses in malignant-cell-associated disease research, leading to potential errors in findings [11]. Uncertainty quantification of the estimated malignant cell fraction is thus essential, as is the quantification of prediction accuracy.

Uncertainty in malignant cell fraction estimation can be quantified through prediction intervals, which provide a range within which the true cell-type composition is likely to fall with a high probability [12, 13]. An ideal procedure for generating prediction intervals should satisfy two properties. The first property is validity [14]. It should provide valid coverage in finite samples without making strong distributional assumptions, such as normality. The second property is discrimination [12]. The predicted intervals should be as narrow as possible at each point in the input space so that the predictions will be informative. When the data is heteroscedastic, getting valid but narrow prediction intervals requires adjusting the lengths of the intervals according to the local variability at each query point in the predictor space.

RNA-Sieve [9] and MEAD [7] are two statistical methods that have been proposed recently that can be used to estimate cell-type proportions and, in the meantime, quantify the uncertainties of the estimated cell proportions. RNA-Sieve [9] is a likelihood-based deconvolution method. It assumes that the estimates of cell-type fractions are normally distributed around the true fractions. Meanwhile, the errors arising from the gene expression profile and observed bulk gene expressions are independent. Therefore, the confidence intervals of the cell proportions can be calculated through likelihood estimation. However, these assumptions may not hold consistently in practice, as gene expression levels within samples (either bulk or single-cell) often exhibit inter-gene dependencies due to coregulation mechanisms [15]. MEAD [7], another statistical inference approach, incorporates a gene-gene dependency structure to improve the accuracy of cell proportion estimates. MEAD asserts that the estimated proportions follow asymptotic normal distributions, with solutions constrained to non-negative values. While MEAD considers the correlation across different genes, the assumption that individuals in the bulk and reference data are from the same population may not hold universally, especially in contexts like cancer research, where gene expression levels vary greatly in different populations. Moreover, the dependence matrix used in MEAD is highly dependent on the choice of bulk samples and cannot be generated when there is only one single bulk sample to decompose.

In this study, we introduce DeepDeconUQ, a deep learning model that is distribution-agnostic and designed to estimate prediction intervals for malignant cell compositions in bulk RNA-seq data. DeepDeconUQ trains a neural network on simulated bulk RNA-seq data, avoiding parametric assumptions about bulk gene expression distributions. Through conformalized quantile regression [14], it provides both valid and precise prediction intervals for malignant cell fractions. Specifically, DeepDeconUQ employs scRNA-seq data to simulate artificial bulk RNA-seq datasets with predefined malignant cell proportions. These simulated datasets are then used to train a quantile regression neural network, which predicts the lower and upper bounds of malignant cell proportions in new cancer tissue samples. Following this, a conformal prediction process is applied to a separate calibration dataset of artificial bulk RNA-seq to adjust the intervals generated by the neural network. This conformalization step ensures that the estimated malignant cell proportions achieve stronger coverage guarantees. Benchmarking with both simulated and real datasets demonstrates that DeepDeconUQ surpasses existing methods in performance and remains robust against perturbations in gene expression levels. By leveraging scRNA-seq data, employing deep neural networks, and utilizing conformalized quantile regression, DeepDeconUQ achieves superior performance in cancer cell deconvolution analysis with uncertainty quantification.

## Results

### Methods overview

**Fig 1:**
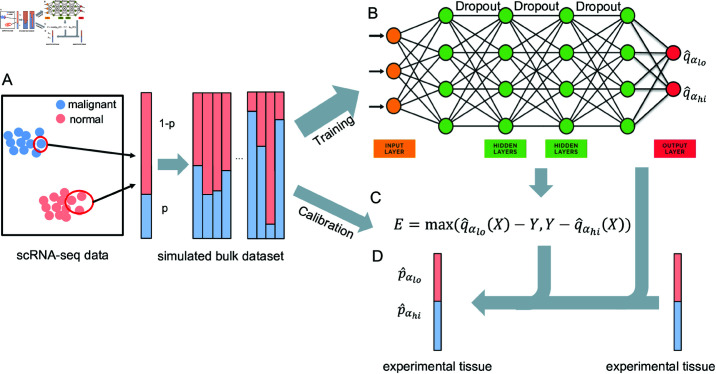
Overview of DeepDeconUQ. **A**: Constructing simulated bulk RNA-seq samples with different fractions of malignant cells. *p* is the fraction of malignant cells in a simulated bulk sample. **B**: Model structure used to train DeepDeconUQ. It consists of four fully connected layers with dropout layers. Seventy percent of the simulated data are used for training. The output is two quantile functions at a given significance level α. **C**: Conformity scores are calculated on the remaining 30% of the simulated dataset. **D**: Estimating the prediction interval of malignant cells from a real bulk sample. The trained model is used to calculate the lower and upper bounds, and the conformity scores are used to adjust the quantiles, which finally outputs the prediction interval $\left\{{\hat{p}}_{\alpha_{lo}},{\hat{p}}_{\alpha_{hi}}\right\}$.

Fig 1 provides a schematic representation of DeepDeconUQ. The framework begins with single-cell RNA sequencing (scRNA-seq) datasets, where the cells from each subject are assumed to have labeled cell types (malignant or normal) and known gene expression profiles. The scRNA-seq data is a gene expression matrix where each row is a single cell sample, and each column is a gene. To simulate bulk RNA-seq data, first, we randomly select certain numbers of malignant and normal cells with replacement. Second, the bulk gene expression profile can be generated by summing up the gene expression values of the selected cells (Fig 1A). These processes are repeated many times to generate a large number of simulated bulk sequencing data. These simulated bulk RNA-seq datasets are then divided into two disjoint groups: a training set and a calibration set. Specifically, 70% of the data is randomly selected for training a highly accurate quantile function, while the remaining 30% is reserved for conformal calibration. After the TF-IDF transformation and MinMax normalization, the trained model uses bulk RNA-seq data *x* and a predefined significance level α as input and outputs predictions of the lower and upper bounds for malignant cell fractions, $\left\{{\hat{q}}_{\alpha_{lo}}(x),{\hat{q}}_{\alpha_{hi}}(x)\right\}$ (Fig 1B). Following model training, the calibration set is employed to compute conformity scores using Eq 10. The adjustment minimizes both the risk of overly conservative predictions (over-coverage) and the potential for overly narrow intervals that miss true values (under-coverage) (Fig 1C). Finally, for a real bulk sample, DeepDeconUQ firstly uses the neural network to get an estimate of the prediction interval and then makes use of the conformity score to adjust the prediction interval $\hat{C}(X_{n+1})$ (Fig 1D). This prediction interval provides a measure of uncertainty, offering a more reliable estimate of the malignant cell fractions within a bulk RNA-seq sample.

Our model was constructed using artificial bulk RNA-seq samples and evaluated through the leave-one-out cross-validation. The evaluation is based on validity and discrimination. For validity, we check the coverage rate, defined as the frequency of true malignant cell fraction within the prediction interval of the testing dataset (see Eq 1). For discrimination, we use the average length of prediction interval of the testing datasets as an evaluation metric (see Eq 2).

$$Coverage=\frac{1}{n}\sum_{i=1}^{n}1({\hat{p}}_{i,\alpha_{lo}}\leq y_{i}\leq {\hat{p}}_{i,\alpha_{hi}}),$$ (1)

$$L_{avg}=\frac{1}{n}\sum_{i=1}^{n}\left|{\hat{p}}_{i,\alpha_{hi}}-{\hat{p}}_{i,\alpha_{lo}}\right|,$$ (2)

where *y*_*i*_ is the true malignant cell fraction of the *i*th sample in the testing dataset. ${\hat{p}}_{i,\alpha_{lo}}$ and ${\hat{p}}_{i,\alpha_{hi}}$ are the corresponding lower and upper bounds of the *i*th sample’s prediction interval. *n* is the total number of samples in the testing dataset, and 1(*x*) is an indicator function of 1 when *x* is true and 0 otherwise.

For each subject, we generated the simulated bulk datasets as described in the Dataset simulation subsection separately. Leave-one-out cross-validation was used to evaluate model performance across subjects during simulation. Specifically, we selected one of the *k* artificial bulk RNA-seq datasets as the testing dataset, while the remaining *k*–1 datasets served as the training set. This process was repeated *k* times to fully evaluate the performance of our model. For real-world dataset applications, we aggregated all *k* artificial bulk RNA-seq datasets to train a unified model, which was subsequently validated using real data.

### DeepDeconUQ outperforms other methods for estimating the prediction interval of malignant cell fraction

To assess the performance of DeepDeconUQ, we conducted a comparative analysis against two alternative methods, RNA-Sieve (v. 0.1.4) [9] and MEAD (v. 1.0.1) [7], both of which have been proposed in the literature to quantify uncertainties in estimated cell-type proportions. This evaluation was performed on both simulated and real bulk RNA-seq datasets. Since RNA-Sieve and MEAD are statistical inference methods and do not include a step for simulating artificial bulk RNA-seq datasets for model training, we utilized the scRNA-seq data directly as the reference for these methods. The same scRNA-seq data were also employed to generate the synthetic bulk RNA-seq datasets for DeepDeconUQ. All benchmarking methods were executed using their default configurations, ensuring a consistent basis for comparison. Additionally, the methods were evaluated on identical test datasets, which were kept separate from the training datasets used to develop the models.

**Fig 2:**
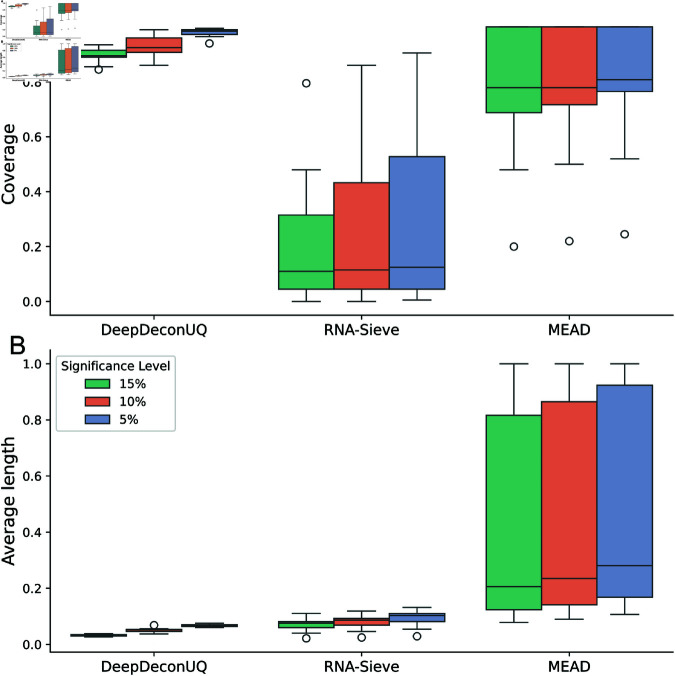
DeepDeconUQ outperforms other methods in predicting malignant cell type prediction interval on AML simulated bulk RNA-seq datasets. Boxplots of coverage (**A**) and average prediction interval length (**B**) on 15 AML simulated bulk RNA-seq datasets. Coverage is defined as the proportion of instances in which the true fraction of malignant cells falls within the prediction interval for the testing dataset. The average length represents the mean length of the prediction intervals across the testing datasets. Each bar in the boxplot comprises 15 data points, each corresponding to one of 15 simulated AML datasets. Significance levels are indicated with different colors.

Fig 2 presents boxplots illustrating coverage and average prediction interval lengths for 15 simulated bulk RNA-seq datasets at three significance levels (15%, 10%, and 5%). Although RNA-Sieve maintains relatively narrow prediction intervals, it often fails to meet the coverage criterion across the datasets, indicating a tendency toward marked undercoverage. This suggests that RNA-Sieve’s intervals may be too narrow to reliably contain the true malignant fraction. In contrast, MEAD achieves the coverage criterion for some datasets but exhibits considerable variability in prediction interval lengths, with some interval lengths extending beyond 0.6. Such substantial intervals lead to overcoverage, reducing interpretability by producing intervals that are too broad to offer precise estimates. DeepDeconUQ demonstrates superior performance across all three methods on the simulation datasets, consistently satisfying the coverage requirement while maintaining tight prediction intervals. This performance advantage is attributed to two primary factors: first, the neural network’s effective quantile learning enables it to meet the coverage criterion; second, the well-trained model generates low conformity scores on the calibration set, ensuring that the quantile of these scores remains sufficiently small to yield narrow prediction intervals.

**Fig 3:**
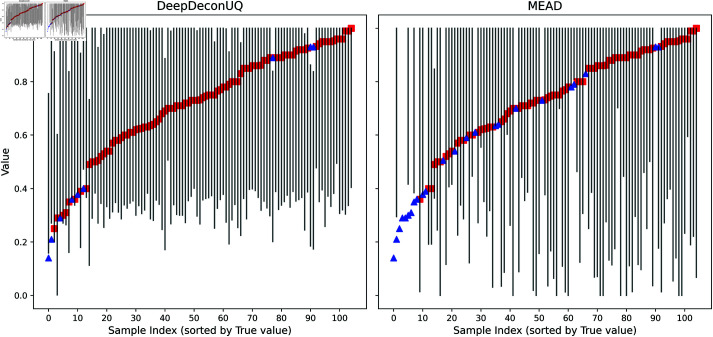
Visualization of prediction intervals on the real primary dataset of DeepDeconUQ and MEAD at α = 0.05 (95% confidence level). Comparison of uncertainty intervals generated by DeepDeconUQ (left) and MEAD (right) methods. Each vertical line represents the prediction interval (lower to upper bound) for an individual sample, with samples sorted by their true malignant fraction values in ascending order along the x-axis. The true values are marked with either red squares (when contained within the prediction interval) or blue triangles (when falling outside the prediction interval).

We further evaluated the performance of these three methods on real AML datasets, including ‘primary,’ ‘recurrent,’ and ‘BeatAML’ samples, one real Neuroblastoma dataset, and one real HNSCC dataset. As illustrated in Table 1, RNA-Sieve consistently has the worst performance, with its average prediction interval length fixed at 1.0, indicating it predicts 0.0 as the lower bound and 1.0 as the upper bound for every real sample. This likely stems from RNA-Sieve’s limitations in handling gene expression data sourced from diverse sequencing protocols. Consequently, while RNA-Sieve can provide an estimate of malignant cell fraction, the results lack reliability. MEAD, conversely, accounts for variations in sequencing depth and tissue sample size, thus yielding relatively robust performance on real datasets. DeepDeconUQ demonstrates an even higher capability by addressing batch effects and sequencing biases via TF-IDF transformation and Min-Max normalization, achieving superior performance relative to MEAD, with more consistent coverage and narrower prediction intervals across the real datasets. Fig 3 depicts the prediction intervals generated by DeepDeconUQ and MEAD on the real primary dataset at α = 0.05 (95% confidence level). Given that RNA-Sieve consistently generated maximum-width prediction intervals (0.0-1.0) on real AML datasets, we restricted our visualization analysis to DeepDeconUQ and MEAD. The visualization clearly demonstrates that MEAD failed to encompass several real samples with true malignant cell fractions in the range of 0.5-0.8, whereas DeepDeconUQ successfully captured all samples within this range. Although both DeepDeconUQ and MEAD exhibited coverage failures for samples with true malignant cell fractions below 0.4, DeepDeconUQ demonstrated superior performance with significantly fewer coverage failures in this lower range. Results for other significance levels can be accessed in Figs A and B in S1 text. It should be noted that the malignant cell fractions given by flow cytometry most likely deviate from a true fraction of malignant cells, resulting in under coverage compared to the prespecified coverage levels, which is expected. Despite these caveats, the results show that the coverages of the prediction intervals from DeepDeconUQ are generally higher than those from MEAD, while the lengths of the prediction intervals from DeepDeconUQ are shorter than those based on MEAD.

**Table 1:** DeepDeconUQ outperforms other methods in predicting malignant cell type prediction interval on real cancer bulk RNA-seq datasets. Coverage and average prediction interval length (L${\;}_{avg}$) are shown under different significance levels on three real AML bulk RNA-seq datasets (‘primary,’ ‘recurrent,’ and ‘BeatAML’), one real Neuroblastoma dataset and one real HNSCC dataset.

Methods	Dataset	15%		10%		5%	
		Coverage	L${\;}_{avg}$	Coverage	L${\;}_{avg}$	Coverage	L${\;}_{avg}$
RNA-Sieve	primary	1.0	1.0	1.0	1.0	1.0	1.0
	recurrent	1.0	1.0	1.0	1.0	1.0	1.0
	beat	1.0	1.0	1.0	1.0	1.0	1.0
	Neuroblastoma	1.0	1.0	1.0	1.0	1.0	1.0
	HNSCC	1.0	1.0	1.0	1.0	1.0	1.0
MEAD	primary	0.667	0.553	0.705	0.630	0.771	0.738
	recurrent	0.676	0.520	0.706	0.591	0.735	0.694
	beat	0.496	0.386	0.544	0.433	0.663	0.515
	Neuroblastoma	0.333	0.211	0.397	0.224	0.428	0.231
	HNSCC	0.139	0.035	0.270	0.059	0.283	0.062
DeepDeconUQ	primary	0.800	0.434	0.876	0.572	0.912	0.662
	recurrent	0.824	0.606	0.853	0.604	0.882	0.685
	beat	0.592	0.409	0.730	0.554	0.781	0.611
	Neuroblastoma	0.349	0.280	0.460	0.293	0.556	0.309
	HNSCC	0.361	0.081	0.433	0.103	0.635	0.151

Additionally, We further compared coverages of the prediction intervals based on DeepDeconUQ and MEAD using McNemar’s statistical test [16]. We also compared the lengths of the prediction intervals based on DeepDeconUQ and MEAD using the Wilcoxon signed-rank test. For the coverage analysis, each sample in the dataset was assigned a label of 1 if its true malignant cell fraction fell within the predicted interval; otherwise, it was labeled as 0. This approach enabled the generation of binary outcome pairs for each sample between DeepDeconUQ and MEAD, thereby providing paired nominal data suitable for McNemar’s statistical test. Furthermore, we aggregated all samples across the three AML datasets into a consolidated dataset to perform a statistical assessment of this unified sample set. The resulting p-values from McNemar’s test are $1.4035\times 10^{-6}$, $1.2438\times 10^{-13}$, and $1.3977\times 10^{-8}$ at significance levels 15%, 10%, 5%, respectively. Moreover, the p-value of the Wilcoxon signed-rank test on the prediction lengths are $3.33\times 10^{-6}$, $9.75\times 10^{-9}$, and 0.0013 at the same significance levels. These findings underscore a statistically significant performance distinction between DeepDeconUQ and MEAD.

We also tested DeepDeconUQ’s performance on two other cancer types, neuroblastoma and head and neck squamous cell carcinoma (HNSCC), as evaluated in DeepDecon (see Figs C and D in S1 text). DeepDeconUQ consistently achieved the highest coverage and the narrowest prediction intervals across all three datasets at different significance levels. The results are presented in Table 1. Moreover, DeepDeconUQ is also robust in complex tumor microenvironments (TME) when tested with epithelial datasets (see Fig E in S1 text).

### DeepDeconUQ is robust to gene expression perturbations

**Fig 4:**
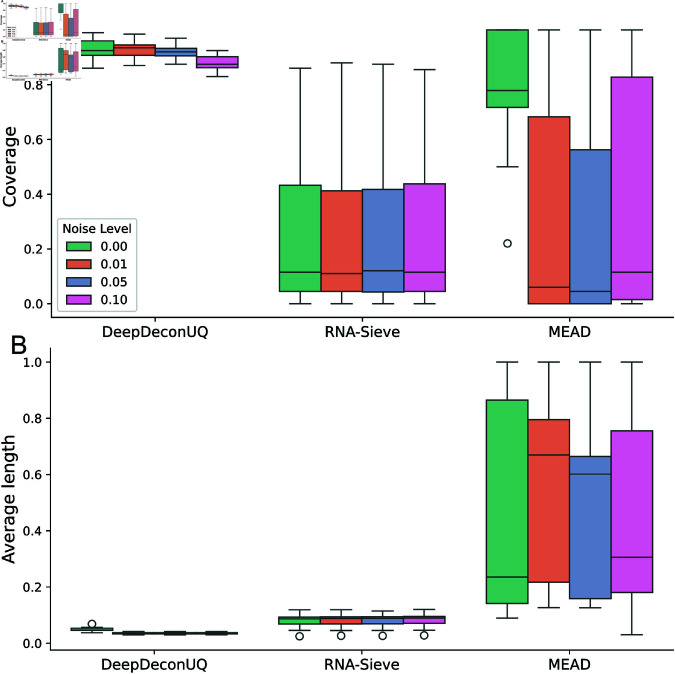
DeepDeconUQ is robust to gene expression perturbations. Boxplots of coverage and average prediction interval length on 15 AML simulated bulk RNA-seq datasets under different noise levels. We added random noise generated from a Gaussian distribution with zero mean and variance that equals λ(λ=0.01,0.05,0.1) times the gene expression level for each gene in each sample. Each bar contains a total of 15 points, representing 15 separate AML datasets. The color represents different levels of noise level λ. Significance level α=0.1.

In the Methods section, we discussed how perturbations in bulk RNA-seq gene expression data can affect the accuracy of the estimation algorithms. Fig 4 and Figs F and G in S1 text illustrate the impact of various perturbation levels on the performance of these methods under different significance levels. For RNA-Sieve, the performance remains comparable to prior results without noise interference, with the prediction interval coverage consistently low. For MEAD, increasing noise levels results in decreased coverage and increased variability in the intervals. In the case of DeepDeconUQ, while coverage decreases as noise levels rise, the majority of coverage values still meet the required threshold. Notably, the average length of DeepDeconUQ’s prediction intervals remains stable across different noise levels. DeepDeconUQ achieves the highest coverage and smallest average interval length across all methods under various noise conditions, demonstrating its robustness to expression perturbations.

### Ablation study

To understand the contribution of key architectural components to model performance, we conducted a systematic ablation study. We focused on two critical elements: conformal calibration and TF-IDF transformation. Quantile regression was preserved throughout this analysis as it provides the fundamental mechanism for generating lower and upper prediction interval bounds.

In the conformal calibration ablation experiment, we eliminated the calibration phase and allocated the entire training dataset to neural network training. For the TF-IDF transformation ablation, we removed this feature engineering step while retaining MinMax normalization, which is essential for stabilizing gradient-based optimization in deep learning frameworks.

**Fig 5:**
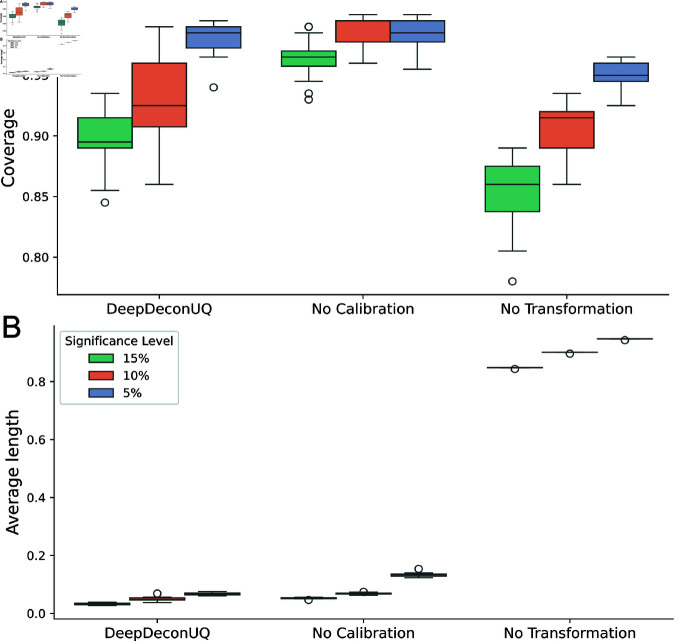
Ablation study of DeepDeconUQ. DeepDeconUQ is the original model. No Calibration removes the calibration part of the DeepDeconUQ model and uses all the training data to train the neural network. No Transformation removes the TF-IDF transformation and uses MinMax normalization for data preprocessing. Each point in the boxplot is an artificial bulk RNA-seq dataset.

The result is shown in Fig 5. When conformal calibration was removed, DeepDeconUQ demonstrated systematic over-coverage with expanded interval widths compared to the original implementation. This finding confirms that conformal calibration plays a crucial role in optimizing prediction intervals by balancing coverage precision and interval width. The elimination of TF-IDF transformation had more pronounced consequences, resulting in a markedly degraded performance characterized by insufficient coverage (substantially below prescribed confidence levels) and wider prediction intervals. The severity of this performance deterioration highlights the fundamental importance of TF-IDF transformation in enabling effective neural network learning.

Collectively, these ablation experiments validate the necessity of both components in the DeepDeconUQ architecture, with each contributing significantly to the model’s overall predictive capabilities and uncertainty quantification accuracy.

### Time and memory usage

DeepDeconUQ was trained and tested on a High-Performance-Cluster (HPC) with a xeon-2640 6-core CPU node. It is the only algorithm that requires the generation of *in silico* training data, which takes 20 min for 3000 samples with a peak memory usage of 10 GB. Additionally, it took ~20 min to train a model and took ~3 s to predict on one bulk tissue.

## Discussion

DeepDeconUQ is an advanced deep neural network-based algorithm designed to leverage single-cell RNA sequencing (scRNA-seq) data to generate prediction intervals for malignant cancer cell fractions. Building on our earlier method, DeepDecon, DeepDeconUQ retains all its foundational advantages, such as the ability to automatically extract complex nonlinear features within its hidden layers and to accurately estimate the quantile function by integrating a comprehensive input of genes ( $\sim 10^{4}$). To address intrinsic variability in RNA-seq data, DeepDeconUQ employs TF-IDF transformation and Min-Max normalization, which enables it to yield prediction intervals that account for both biological and technical sources of noise. Additionally, it utilizes a calibration dataset to fine-tune the prediction interval, effectively mitigating risks of overcoverage and undercoverage. Integrating training and calibration datasets in DeepDeconUQ represents a significant advancement in malignant cancer cell fraction estimation, allowing for more accurate and interpretable predictions. By leveraging quantile regression and conformal inference, DeepDeconUQ not only enhances confidence in the malignant cell prediction interval results but also facilitates the application of the method to real-world datasets with minimal adjustments. The framework’s ability to generate reliable uncertainty estimates positions DeepDeconUQ as a valuable tool for the analysis of bulk RNA-seq data, particularly in contexts where precise quantification of cell type proportions is critical for downstream analyses and clinical decision-making.

While DeepDeconUQ can achieve good performance on AML cancer tissues, we note that this method still has limitations. First of all, the quality of training data is very important. DeepDeconUQ is a neural network-based method, which means it needs a large amount of data to train. Currently, we use single-cell data from 15 AML subjects to construct simulation bulk RNA-seq datasets. If the number of subjects is small or the single-cell data is dominated by one specific cell type, DeepDeconUQ can learn less information from the data and cannot generalize and represent the latent features well. In theory, the UQ approach may also work for previous decomposition methods with or without single-cell data, provided we have sufficient bulk RNA-seq data with corresponding malignant cell fractions. A critical prerequisite is that these annotated fractions should span the complete range from 0.0 to 1.0. However, from a practical perspective, such comprehensively annotated bulk RNA-seq datasets remain scarce. Secondly, experimental bias and noise can greatly affect the estimate performance, even though we take different ways such as TF-IDF transformation and Min-Max normalization to mitigate batch effects and bias. The complexity and difficulties of real RNA-seq can still affect DeepDeconUQ’s performance. Thirdly, DeepDeconUQ can only estimate the prediction interval of malignant cell fraction. In practice, tissues usually consist of multiple cell types, and some tissues even contain unknown sub-cell types.

We plan to further improve the performance and applicability of DeepDeconUQ by implementing several key modifications to the existing methodology. Firstly, we want to extend DeepDeconUQ’s capacity to include multiple cell types or subtypes. The current method avoids the statistical complexity of handling multivariate prediction regions, which are required when deconvolving bulk RNA-seq data into more than two cell types. Prediction regions, unlike univariate intervals, must account for dependencies between cell type proportions (e.g., sum-to-one constraints and correlations), necessitating advanced methods like multivariate conformal prediction. Secondly, DeepDeconUQ’s capability to detect technical bias and diverse sequencing protocols should be improved. In addition to current normalization processing, methods like autoencoder [17, 18], transfer learning [19] and transformers [20] can be used to generate latent embeddings to reduce these biases (see Fig H in S1 text). Thirdly, the current DeepDeconUQ model takes all genes into account. Whether selective incorporation of cell type-specific genes could enhance prediction accuracy is an interesting topic. To investigate this issue, we selected differentially expressed genes between normal and malignant cells using MAST [21], a widely used method for single cell differential gene analysis. DeepDeconUQ was trained and validated based on the selected genes and the detailed results are given in Fig I and Table A in S1 text. The preliminary study shows that gene selection does not markedly impact the performance of DeeDeconUQ. More complete and extensive studies on the impacts of gene selection using other software packages on the performance of DeepDeconUQ will be studied in the future.

## Materials and methods

### Datasets

To initially train and test DeepDeconUQ, we utilized simulated datasets derived from Acute Myeloid Leukemia (AML) single-cell data previously used in DeepDecon [22]. The single-cell AML datasets were downloaded from Gene Expression Omnibus (GEO) with accession number GSE116256 [23]. We selected 15 subjects, totaling 38,410 cells, to simulate artificial bulk RNA-seq datasets, employing the same preprocessing and simulation procedures established in DeepDecon. Preprocessing of scRNA-seq data followed the workflow of Scanpy (v.1.7.2), a widely-adopted Python package for single-cell gene expression analysis [24]. Initially, cells with fewer than 500 detected genes and genes expressed in fewer than five cells were filtered out (Fig J in S1 text). Further, gene expression count matrices were processed to remove extreme outliers (Table B in S1 text). Gene expression values were normalized using Scanpy’s ‘normalize_total’ function to ensure uniform total counts across cells. This will mitigate discrepancies arising from varying library sizes. This produced a normalized matrix of all filtered cells and genes, ready for the generation of simulated bulk data. Ultimately, 30,000 simulated bulk samples (2,000 per subject) were generated for training and testing DeepDeconUQ.

We further assessed DeepDeconUQ using real AML bulk RNA-seq datasets. Real AML data were collected from the GDC Data Portal (https://portal.gdc.cancer.gov/) with the project name “TARGET-AML". The AML samples were further divided into primary and recurrent AML categories according to different cancer stages. As a result, there were a total of 117 primary AML samples and 38 recurrent AML samples. For these bulk RNA-seq datasets, ground-truth cancer cell fractions via flow cytometry are available. Additionally, an independent real AML dataset, “BeatAML" [25], was collected from cBioportal [26]. “BeatAML" contains a total of 451 bulk RNA-seq samples and 300 of them have corresponding ground-truth cancer cell fractions. This dataset used the “SureSelect" sequencing platform, which is different from the sequencing platform for the single-cell data in “TARGET-AML" dataset (Table C in S1 text). The inclusion of these diverse datasets allowed us to evaluate DeepDeconUQ’s performance across different sequencing platforms and data sources.

To test DeepDeconUQ’s performance on other cancer tissues, we also collected 19,173 single cells from 9 neuroblastoma cancer patients [27] and 184,868 single cells from 27 Head and neck squamous cell carcinoma (HNSCC) cancer patients [28]. They were used to simulate artificial RNA-seq bulk samples to build and evaluate DeepDeconUQ. Additionally, a real neuroblastoma bulk RNA-seq dataset consisting of 99 bulk RNA-seq samples with known cancer cell fractions was collected from cBioportal [26] and another real HNSCC bulk RNA-seq dataset, ‘TCGA-HNSC’, consisting of 518 bulk RNA-seq samples with known cancer cell fractions was collected from LinkedOmics [29]. These two real datasets were used for testing. Moreover, the above datasets have the knowledge of malignant and normal cells. However, in practice, cancer tissues usually exhibit a complex tumor microenvironment (TME). A total of 18,062 single cells derived from four individuals were collected [30], It contains epithelial cells (tumor), T-cells, B-cells, plasma cells, macrophage, fibroblast cells, and so on. Experiments were conducted to test the capacity of DeepDeconUQ to estimate epithelial cell proportion regarding heterogeneity.

### Generating artificial bulk RNA-seq datasets

To generate artificial bulk RNA-seq samples, we used the previously described scRNA-seq datasets, simulating each sample with predetermined malignant cell fractions for training the DeepDeconUQ model. Specifically, for each artificial bulk sample, we set a fixed total cell count, *N*, and a malignant cell number *n*_*m*_ was randomly sampled from a uniform distribution between 0 and *N*. Subsequently, *n*_*m*_ malignant cells and $N\;-\;n_{m}$ normal cells were randomly drawn from the same scRNA-seq dataset. If the available malignant or normal cells were fewer than *n*_*m*_ or *N*−*n*_*m*_, respectively, cells were sampled with replacement, meaning that each cell was uniformly drawn from all single cells in the dataset; otherwise, cells were sampled without replacement to ensure no duplicates. Importantly, cells from different subjects (i.e., individuals) were not combined within a single artificial sample to maintain individual-specific gene expression profiles. This principle was motivated by two reasons. Firstly, the aim was to safeguard within-subject relationships among genes by preserving the unique gene expression patterns inherent to each subject. Secondly, the intention was to capture the variability between subjects, commonly referred to as cross-subject heterogeneity [8]. After generating an artificial bulk sample by summing the expression values of all selected cells, it was labeled according to the malignant cell fraction, *n*_*m*_/*N*. This process was repeated for each scRNA-seq dataset, resulting in a corresponding artificial bulk RNA-seq dataset with *T* samples, each tagged with a known malignant cell proportion. Here, we set N=3,000 and *T* = 200, consistent with the configuration in DeepDecon [22]. This sampling strategy serves as a substantial data generation resource for training and evaluating DeepDeconUQ.

### Data processing

Before training, the artificial bulk RNA-seq samples were preprocessed to ensure alignment between training and prediction data. Only genes present in both the training and testing datasets were retained, and genes with low expression variance (below 0.1) were excluded. To further standardize the data, a TF-IDF transformation was applied to the raw RNA-seq count matrix. This transformation, commonly used in information retrieval and text mining [31, 32], starts by calculating the ‘term frequency (TF)’ for each gene in each sample by normalizing the gene expression profile (see Eq 3). The ‘inverse document frequency (IDF)’ was then calculated by dividing the total number of bulk samples by the total gene expression values of the gene across all samples (see Eq 4), followed by log-transformation and multiplication by the TF value. The TF-IDF transformation weights genes with lower expression levels more heavily, which helps to adjust for the imbalanced expression levels across genes [33].

$$TF(X_{i,j})=\frac{X_{i,j}}{\sum_{j}X_{i,j}},$$ (3)

$$IDF(G_{j})=\log\left(\frac{T}{\sum_{i}X_{i,j}}+1\right),$$ (4)

where *X*_*i*,*j*_ is the expression level of the *j*th gene in the *i*th sample, *G*_*j*_ indicates the *j*th gene, and *T* is the number of bulk samples.

Let $X^{\prime }$ denote the gene expression matrix after TF-IDF transformation. A MinMax normalization was applied to the resulting expression matrix $X^{\prime }$ to scale the expression values to the [0, 1] range (see Eq 5). This is a common practice in deep learning models that use gradient-based optimization algorithms [8, 17].

$$X_{i}^{norm}=\frac{X_{i}^{\prime }-\min(X_{i}^{\prime })}{\max(X_{i}^{\prime })-\min(X_{i}^{\prime })},$$ (5)

where $X_{i}^{\prime }$ is the *i*th row of $X^{\prime }$ and $X_{i}^{norm}$ is the *i*th row of the resulting expression matrix after the MinMax transformation.

TF-IDF transformation and MinMax normalization are important steps in ensuring the quality and consistency of the data used to train deep learning models. Although the input datasets varied between platforms and protocols, we utilized the same processing workflow to make it easy to apply DeepDeconUQ to other datasets.

### DeepDeconUQ

#### Problem formulation.

Suppose we are given *n* bulk RNA-seq gene expression samples $\{(X_{i},Y_{i}){\}}_{i=1}^{n}$, where $X_{i}\in {\mathbb{R}}^{p}$ represents the *i*th bulk RNA-seq gene expression vector with *p*>0 features (genes) and $Y_{i}=(y_{i},1\;-\;y_{i})$ is the corresponding *i*th cell fraction vector of malignant and normal cells. Our aim is to construct a distribution-agnostic prediction interval $\hat{C}(X_{n+1})$ that contains the malignant cell fraction *y*_*n* + 1_ for a new bulk RNA-seq sample *X*_*n* + 1_. Specifically, given a desired significance level α, the prediction interval $\hat{C}(X_{n+1})$ is likely to contain the true malignant cell fraction vector *y*_*n* + 1_ with a user-specified coverage probability 1−α:

$$\mathbb{P}\{y_{n+1}\in \hat{C}(X_{n+1}))\}\geq 1-\alpha ,$$ (6)

for any joint distribution *P*_*XY*_ and any sample size *n* (Eq 6). Meanwhile, the estimated prediction interval $\hat{C}(X_{n+1})$ should be as narrow as possible while achieving the desired coverage level.

#### Quantile regression.

Methods like DeepDecon [22] formulate the problem as a regression task, typically addressed using variations of non-negative least squares or more advanced machine learning methodologies. The estimation of cell type proportions is often solved by minimizing squared residuals over the *n* training points $\{(X_{i},Y_{i}){\}}_{i=1}^{n}$ (see Eq 7):

$$\hat{\mu }(x)=\mu (x;\hat{\theta }),\;\hat{\theta }={argmin}_{\theta }\frac{1}{n}\sum_{i=1}^{n}(Y_{i}-\mu (X_{i};\theta ){)}^{2}+R(\theta ),$$ (7)

where θ are the parameters of the regression model, μ(x;θ) is the learned regression model, and R(θ) is a regularization module.

Similarly, quantile regression estimates the conditional quantiles of cell type proportions, assuming that the τth conditional quantile is associated with gene expression profiles. A conditional quantile function $q_{\alpha }$ is learned from *n* training samples $\{(X_{i},Y_{i}){\}}_{i=1}^{n}$ at a specified quantile (or significance) level α (see Eq 8).

$${\hat{q}}_{\alpha }(x)=f(x;\hat{\theta }),\;\hat{\theta }={argmin}_{\theta }\frac{1}{n}\sum_{i=1}^{n}\rho_{\alpha }(Y_{i},f(X_{i};\theta ))+R(\theta ),$$ (8)

where f(x;θ) is the quantile regression function and can be learned through neural networks. $\rho_{\alpha }$ is the quantile (pinball) loss [34], defined as,

$$\rho_{\alpha }(y,\hat{y})=\left\{ \begin{array}{c} \alpha (y-\hat{y})\;if\;y-\hat{y}>0,\\ (1-\alpha )(\hat{y}-y)\;otherwise, \end{array}\right.$$ (9)

where *y* and $\hat{y}$ are the observed and predicted cell type fraction, and α∈(0,1) is the corresponding quantile (significance) level. Pinball loss is a skewed transformation of the absolute value function and is commonly used in quantile regression [14].

Given a significance level α, we can get the lower bound and upper bound prediction ${\hat{q}}_{\alpha_{lo}},{\hat{q}}_{\alpha_{hi}}$ through quantile regression. Here, $\alpha_{lo}=\frac{\alpha }{2},\alpha_{hi}=1\;-\;\frac{\alpha }{2}$. Then, $\hat{C}(X_{n+1})=[{\hat{q}}_{\alpha_{lo}},{\hat{q}}_{\alpha_{hi}}]$ can be used as the estimate of the true prediction interval *C*(*X*_*n* + 1_). The simplicity and generality of this approach make quantile regression highly versatile, allowing for the integration of various machine learning techniques to model and learn $q_{\alpha }$ [14, 35, 36].

#### Conformal prediction.

The quantile regression method is widely applicable and often works well in practice, yielding intervals that are adaptive to heteroscedasticity. However, it is not guaranteed to satisfy the validity property when the true prediction interval *C*(*X*_*n* + 1_) is estimated by the prediction interval $\hat{C}(X_{n+1})$. Fortunately, conformal prediction [37] was then brought out to solve this problem. Specifically, split (inductive) conformal prediction [38, 39], which is general and whose computational cost is a small fraction of the full conformal prediction, helps construct prediction intervals that are valid and discriminative. We borrowed the idea from Romano *et al*. [14] and combined DeepDecon with conformal quantile regression (CQR) to obtain valid and discriminative cell fraction prediction intervals on bulk RNA-seq samples. We refer the resulting algorithm as DeepDeconUQ.

The split conformal method begins by splitting the training data into two disjoint subsets: a proper training set $\left\{(X_{i},Y_{i}):i\in I_{1}\right\}$ and a calibration set $\left\{(X_{i},Y_{i}):i\in I_{2}\right\}$. We then apply a neural network to estimate the lower and upper quantile functions, ${\hat{q}}_{\alpha_{lo}}$ and ${\hat{q}}_{\alpha_{hi}}$, as described in Eq 8. This model’s architecture is similar to our previously developed cell fraction estimation framework, DeepDecon [22], and will be further explained in the model structure subsection.

Next, we compute conformity scores that quantify the error made by the prediction interval. The scores are evaluated on the calibration set as follows:

$$E_{i}:=max({\hat{q}}_{\alpha_{lo}}(X_{i})-Y_{i},Y_{i}-{\hat{q}}_{\alpha_{hi}}(X_{i}))\;i\in I_{2},$$ (10)

Finally, given new input data *X*_*n* + 1_, we construct the prediction interval of *Y*_*n* + 1_ as:

$$\hat{C}(X_{n+1})=[{\hat{q}}_{\alpha_{lo}}(X_{n+1})-Q_{1-\alpha }(E,I_{2}),{\hat{q}}_{\alpha_{hi}}(X_{n+1})+Q_{1-\alpha }(E,I_{2})],$$ (11)

where $Q_{1-\alpha }(E,I_{2})$ is the $\left(1-\alpha /2)(1+\frac{1}{\left|I_{2}\right|}\right)$th quantile of $\left\{E_{i}:i\in I_{2}\right\}$. In this context, we select α/2 due to the presence of two distinct cell types within the dataset—malignant and normal—as suggested in multivariate quantile regression [40]. Moreover, Romano *et al*. demonstrated that when conformity scores *E*_*i*_ are almost surely unique, the prediction interval achieves an approximate state of perfect calibration [14].

The specific steps of DeepDeconUQ are given in Algorithm 1.

**Algorithm 1 DeepDeconUQ.**

**Require:** Bulk RNA-seq samples with labels $(X_{i},Y_{i})\in {\mathbb{R}}^{p}\times {\mathbb{R}}^{2}$,

1≤i≤n

Significance level α

Testing bulk sample *X*_*n* + 1_

**Ensure** Cell fraction prediction interval *C*(*X*_*n* + 1_) for *X*_*n* + 1_.

1: Randomly split *n* bulk RNA-seq samples into two disjoint sets, *I*_1_ and *I*_2_.

2: Fit two conditional quantile functions $\left\{{\hat{q}}_{\alpha_{lo}},{\hat{q}}_{\alpha_{hi}}\right\}$ according to Eq 8 on training set *I*_1_

3: Compute conformity scores *E*_*i*_ according to Eq 10 on calibration set *I*_2_

4: Compute $Q_{1-\alpha }(E,I_{2})$, the $\left(1-\alpha /2)(1+\frac{1}{\left|I_{2}\right|}\right)$th quantile of $\left\{E_{i}:i\in I_{2}\right\}$.

5: Compute prediction interval $\hat{C}(X_{n+1})$ according to Eq 11 for *X*_*n* + 1_.

Lei *et al*. advocated for selecting a larger *I*_1_ compared to *I*_2_ to improve the accuracy of estimated quantile functions [41]. Given the size of our training dataset (30,000 simulated samples), we opted for a 7:3 split ratio between the training and calibration sets to optimize the model performance.

#### Model structure.

The main neural network architecture of DeepDeconUQ is similar to DeepDecon, which consists of two main components. The first component consists of four fully connected layers with a dropout regularization between each layer, and the rectified linear unit (ReLU) is used as the activation function in every internal layer. The second component differs from DeepDecon, which uses a softmax function to predict the malignant and normal cell fractions. To reduce the computational cost, instead of fitting two separate neural networks to estimate the lower and upper quantile functions, we replaced the original one-dimensional estimate of the malignant cell fraction with a two-dimensional estimate of the lower and upper quantiles. In this way, most of the network parameters are shared between the two quantile estimators. All model parameters were optimized using the Adam optimization algorithm [42] with a learning rate of 0.0001 and a batch size of 128. The model was trained as a regression task, with the pinball loss (see Eq 9) as the loss function. Hyperparameters that are tested and tuned in DeepDecon were also used in DeepDeconUQ.

### The impact of gene expression perturbations on DeepDeconUQ

To test the model’s robustness to gene expression perturbations, we introduced varying levels of Gaussian noise to the expression levels within the simulated datasets. Specifically, for each gene in each sample, random noise was added, drawn from a Gaussian distribution with a mean of zero. The variance of this noise was proportional to the expression level of each gene, set at λ times the gene expression level, where λ was assigned values of 0.01, 0.05, and 0.1 (see Eq 12). This approach allowed us to systematically examine the model stability and predictive accuracy under controlled levels of expression variability.

$$X_{ij}^{noise}=max(0,X_{ij}+N(0,\lambda X_{ij})),$$ (12)

where *X*_*ij*_ is the gene expression value of gene *j* in simulated bulk sample *i* and λ is the noise level.

Following this processing, we applied the previously trained DeepDeconUQ models to each simulated bulk RNA-seq dataset to estimate the prediction intervals. This enabled us to systematically evaluate the model’s robustness under various gene expression perturbations, providing insights into its stability and reliability in producing accurate intervals when gene expression data is subject to different levels of noise.

## Supporting information

S1 Text Contains data preprocess and additional analysis. Figs A–J with their descriptions, Tables A–C with their descriptions.
(PDF)
