# Supplementary material for: DeepDeconUQ estimates malignant cell fraction prediction intervals in bulk RNA-seq tissue
Source: PLoS Comput Biol. 2025 Jun 4;21(6):e1013133. doi: 10.1371/journal.pcbi.1013133 (PMC12162100; doi:10.1371/journal.pcbi.1013133)
Supplement: S1 Text — Figs A–J with their descriptions, Tables A–C with their descriptions. (PDF) [file pcbi.1013133.s001.pdf]

Supplementary Information
DeepDeconUQ Estimates Malignant Cell Fraction Prediction Intervals in Bulk RNA-seq Tissue

Jiawei Huang[1], Yuxuan Du[1,4], Kevin R. Kelly[3], Jinchi Lv[5], Yingying Fan[5], Jiang F. Zhong *[2] and Fengzhu Sun †[1]

[1]Department of Quantitative and Computational Biology, University of Southern California, Los Angeles, California, United States of America
[2]Department of Basic Sciences, School of Medicine, Loma Linda University, Loma Linda, California, United States of America
[3]Division of Hematology, University of Southern California, Los Angeles, California, United States of America
[4]Department of Electrical and Computer Engineering, University of Texas at San Antonio, San Antonio, Texas, United States of America
[5]Data Sciences and Operations Department, University of Southern California, Los Angeles, California, United States of America

1 Preprocessing of single-cell gene expression data

The AML single cell RNA-seq data used in DeepDeconUQ is the same as the data used in DeepDecon [1]. AML data was obtained from the Gene Expression Omnibus (GEO) under accession number GSE116256 [2]. To ensure data quality, we utilized single-cell RNA sequencing (scRNA-seq) data from subjects with at least 100 normal and 100 malignant cells, respectively (Fig J). This criterion was employed to avoid extreme scenarios in which very few normal or malignant cells were selected. A total of 15 AML subjects were selected. For each subject, we filtered out cells with less than 500 detected genes and genes expressed in less than five cells. The resulting gene expression profile for each subject was further filtered for extreme outliers in gene expression values. The filtering criteria for each subject were given in Table B. Finally, gene expression was normalized to library size by total counts across all genes. This will counteract the effect of different library sizes. Finally, the resulting normalized matrix of all filtered cells and genes was saved for subsequent pseudo bulk data generation. We used the tag 'PredictionRefined' [2] given in the dataset as the final label (malignant/normal) to annotate the cell types. This tag was a manual reclassification of cells by close inspection of mutations/expression profiles.

2 Artificial bulk dataset simulation

The simulated artificial bulk datasets were generated by subsampling within each scRNA-seq subject. Cells from different subjects were not merged into one bulk sample to preserve potential

*Corresponding author: jzhong@llu.edu
†Corresponding author: fsun@usc.edu

correlations between the expression levels of different genes within subjects. The true cell-type proportion for each bulk sample was calculated by dividing the number of single cells with a specific cell type by the total number of cells in the bulk sample.

First, in each bulk scRNA-seq data, N cells from different cell types (malignant/normal) were generated where "1" and "2" correspond to malignant and normal cell types, respectively. Let

$$N = n_1 + n_2, \tag{1}$$

$$f_i = \frac{n_i}{N}, i = 1, 2 \tag{2}$$

where n_i and f_i are the number and fraction of cells of type i, respectively, and N is the total number of cells in one simulated bulk sample. Here n_i was generated uniformly from 0 to N through the python random module [3]. When n_i was determined, n_i cells were sampled from the scRNA-seq gene expression matrix for each cell type i (if n_i is bigger than the total number of cells of type i in one particular subject, the cells were chosen with replacement. Otherwise, the cells were chosen without replacement). Next, the selected single-cell expression profiles for every cell type were aggregated by summing their expression values,

$$G = \sum_i \sum_j X_{ij}, \tag{3}$$

where X_{ij} is the jth gene expression vector of cell type i and G is the final bulk RNA-seq expression profile. Repeating the above steps T times to construct a simulated bulk dataset with T samples. In our simulations, T was chosen as 200 for each subject. Finally, we had 15 simulated bulk RNA-seq datasets, each with 200 bulk samples with known cell type proportions.

3 The influence of feature embedding to DeepDeconUQ

Although batch effects can be mitigated through TF-IDF transformation and Min-Max normalization in DeepDeconUQ, some denoising methods can still be tried to test DeepDeconUQ's performance. One of such is feature embedding.

To test more advanced denoising methods, we employed both classical dimensionality reduction via Principal Component Analysis (PCA) and an advanced transfer learning method, Deep Adaptation Network (DAN) [4]. DAN represents a neural architecture that aligns feature distributions between training and testing domains. DAN uses both the labels and samples of training data and only uses the samples of testing data to generate domain-invariant latent embeddings with theoretical guarantees. It will generate a latent embedding for both the training and testing datasets.

For PCA-based dimensionality reduction, data underwent standard preprocessing (TF-IDF transformation followed by MinMax normalization) before extracting the top 100 principal components as neural network inputs. The DAN implementation faithfully reproduced the methodology described by Long et al. [4], generating latent embeddings for both training and testing datasets that subsequently served as inputs for the neural network training pipeline. All downstream computational processes remained consistent with the original DeepDeconUQ framework.

Performance comparisons revealed distinct trade-offs between methods (Fig H). PCA embeddings demonstrated systematic over-coverage in simulation datasets, with coverage rates exceeding corresponding significance thresholds. Even though it has narrower prediction intervals than baseline DeepDeconUQ. However, when evaluated on real-world datasets, PCA embeddings exhibited reduced coverage relative to the original model, suggesting potential overfitting (Table A). While

achieving coverage rates appropriately aligned with significance levels, DAN embeddings produced substantially wider prediction intervals than the baseline model. We hypothesize that inherent biological heterogeneity across cancer patients presents fundamental challenges to transfer learning in this context, limiting the effectiveness of domain adaptation techniques.

4 Extention to complex tumor microenvironment (TME)

DeepDeconUQ requires the knowledge of malignant and normal cells. However, in practice, cancer tissues usually exhibit a complex tumor microenvironment (TME). There are usually multiple subtypes for either malignant or normal cells. In this case, we can merge the malignant subtypes into one malignant type and the normal subtypes into one normal type. DeepDeconUQ can then be used to estimate the prediction interval of the malignant cell fraction. Specifically, we have collected single-cell data from [5], which comprises scRNA-seq profiles from seven individuals. Following data filtering procedures identical to those applied to the AML dataset (detailed in Section 1), we retained 18,062 single cells derived from four individuals. Cell classifications were established using the 'condition' parameter from Sathe, et al. 2023 [5]. These individuals contain tumor epithelial cells as malignant labels and NK, T-cell, B-cell as normal labels. We subsequently conducted analogous experiments to those performed with the AML dataset. This involved initially constructing synthetic bulk RNA-seq datasets with variable malignant cell proportions based on the available scRNA-seq data. We then trained a DeepDeconUQ model to estimate prediction intervals for malignant cell fractions.

Fig E illustrates the performance of DeepDeconUQ on the epithelial datasets compared to existing methodologies. RNA-Sieve demonstrated a low performance, with coverage probabilities substantially below threshold values across different significance levels, indicating under-coverage. MEAD exhibited complete performance failure on the epithelial dataset, generating prediction intervals approximating 0-1 for all samples and, in some instances, failing to produce valid prediction intervals entirely (returning NA values). In contrast, DeepDeconUQ exhibited superior performance, yielding statistically valid coverage with comparatively narrow prediction intervals. The modest over-coverage observed is likely attributed to the limited training cohort (n=4) and could potentially be mitigated by incorporating additional high-quality datasets.

5 Influence of gene selection on DeepDeconUQ

In the current implementation, DeepDeconUQ utilizes the complete gene set for prediction. We investigated whether selective incorporation of cell type-specific genes could enhance prediction accuracy. To address this question, we employed MAST (Model-based Analysis of Single-cell Transcriptomics) [6] as a differential gene expression analysis tool to evaluate the impact of feature selection on DeepDeconUQ performance.

We applied MAST to identify differentially expressed genes between malignant and normal cell populations within the AML scRNA-seq datasets. MAST takes the AML scRNA-seq datasets as input and runs a likelihood ratio test to get differential genes between two conditions (malignant/normal). This approach yielded $1,414$ genes with adjusted p-values below the significance threshold of 0.05. Subsequently, we executed DeepDeconUQ using only this subset of differentially expressed genes and compared the results against the original implementation utilizing the complete gene set.

Fig I illustrates DeepDeconUQ performance with and without gene selection on simulated AML datasets, while Table A presents corresponding results on real AML datasets. In simulated datasets,

DeepDeconUQ demonstrated comparable performance regardless of gene selection strategy, highlighting the model's inherent robustness. However, in real AML datasets, the considerable heterogeneity of cancer tissue potentially resulted in differential gene signatures that did not effectively generalize across real samples. Consequently, DeepDeconUQ with gene selection exhibited reduced coverage rates at all significance levels when applied to recurrent and beat AML datasets compared to the original implementation.

6 Software comparison and settings

We compared DeepDeconUQ with other deconvolution methods, including MEAD (v. 1.0.1) [7], RNA-Sieve (v. 0.1.4) [8].

For MEAD [7], we installed the R package given in the manuscript and ran it with default settings. In the leave-one-out cross-validation, the single-cell profile was constructed using the single cells of all subjects, excluding the subject itself, while in the real bulk testing data, the single-cell profile was constructed by combining all available single-cell data. Subject information was included in the single-cell reference. Then, we ran MEAD with default settings by following the example provided by the authors.

For RNA-Sieve [8], we executed it by following the example code provided. In the leave-one-out cross-validation, the single-cell profile was constructed by combining the single cells of all subjects, excluding the data itself, while in the real bulk testing data, the single-cell profile was constructed by combining all available single-cell data.

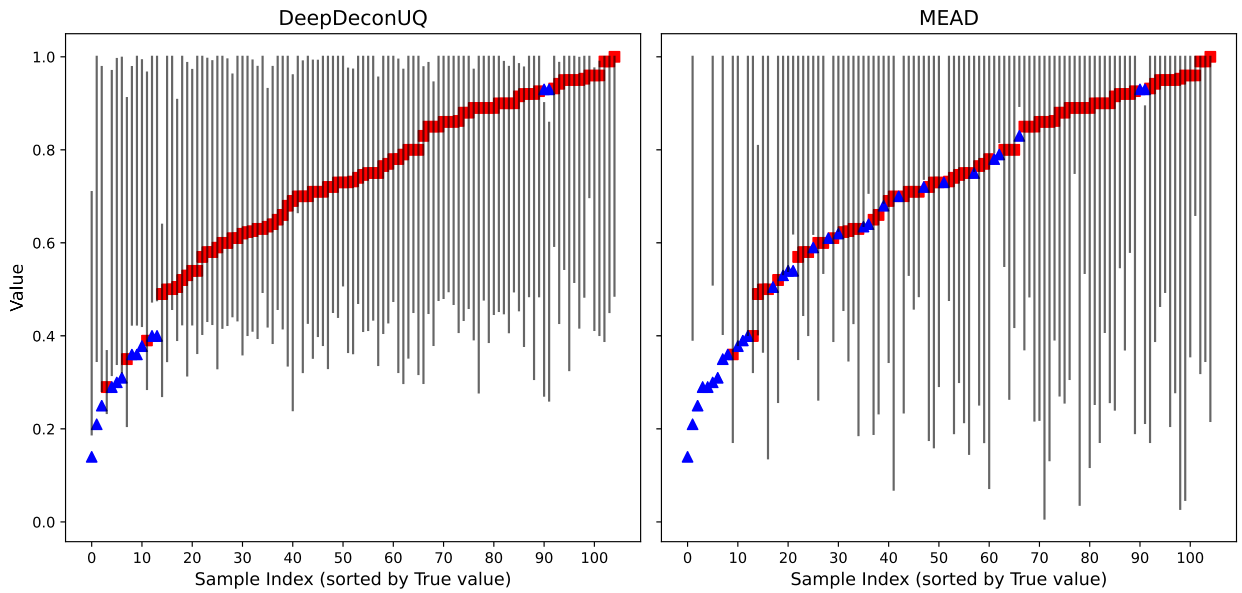

Fig A: **Visualization of prediction intervals on the real primary dataset of DeepDeconUQ and MEAD at $\alpha = 0.10$ (90% confidence level).** Comparison of uncertainty intervals generated by DeepDeconUQ (left) and MEAD (right) methods. Each vertical line represents the prediction interval (lower to upper bound) for an individual sample, with samples sorted by their true malignant fraction values in ascending order along the x-axis. The true values are marked with either red squares (when contained within the prediction interval) or blue triangles (when falling outside the prediction interval).

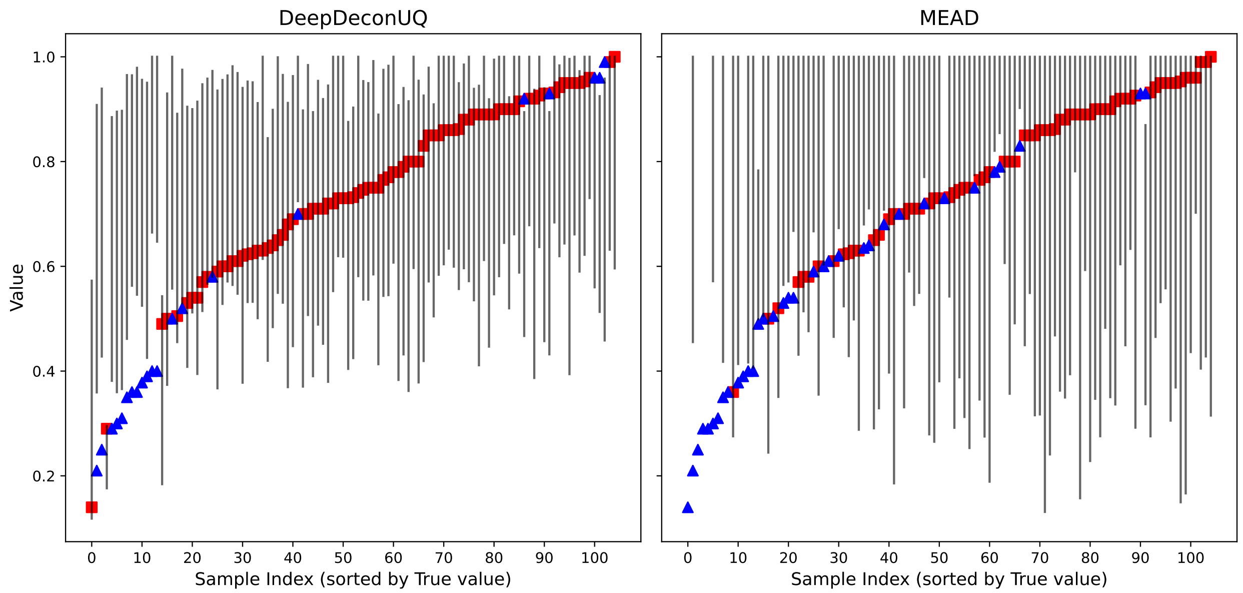

Fig B: **Visualization of prediction intervals on the real primary dataset of DeepDeconUQ and MEAD at $\alpha = 0.15$ (85% confidence level).** Comparison of uncertainty intervals generated by DeepDeconUQ (left) and MEAD (right) methods. Each vertical line represents the prediction interval (lower to upper bound) for an individual sample, with samples sorted by their true malignant fraction values in ascending order along the x-axis. The true values are marked with either red squares (when contained within the prediction interval) or blue triangles (when falling outside the prediction interval).

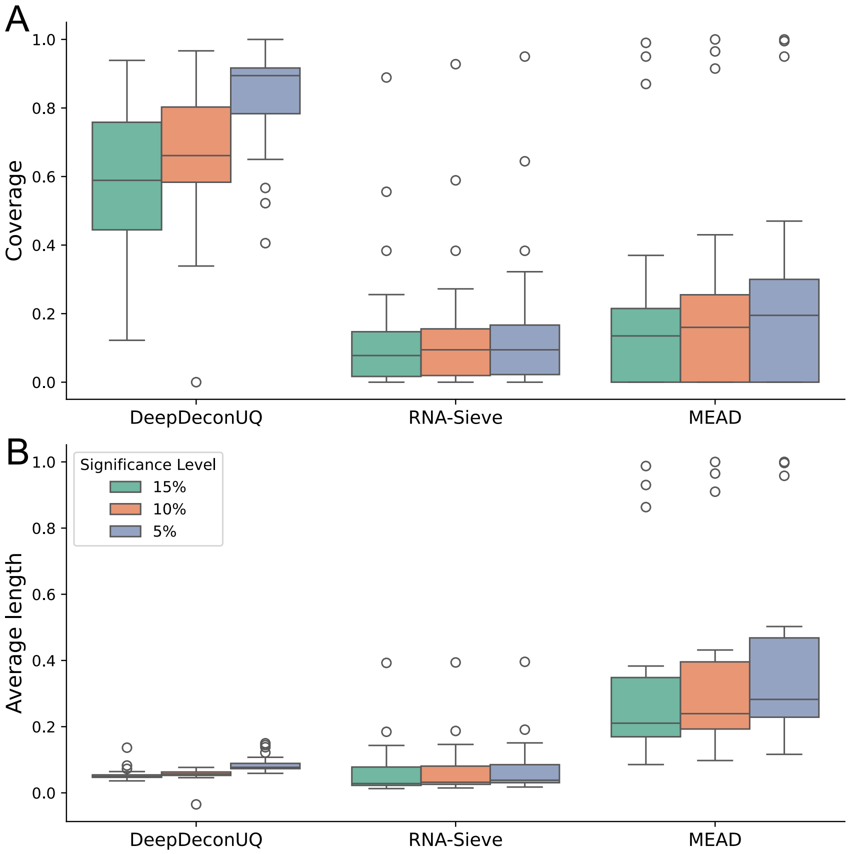

Fig C: **DeepDeconUQ outperforms other methods in predicting malignant cell type prediction interval on simulated HNSCC bulk RNA-seq datasets.** Boxplots of coverage (A) and average prediction interval length (B) on simulated HNSCC bulk RNA-seq datasets. Coverage is defined as the proportion of instances in which the true fraction of malignant cells falls within the prediction interval for the testing dataset. The average length represents the mean length of the prediction intervals across the testing datasets. Significance levels are indicated with different colors.

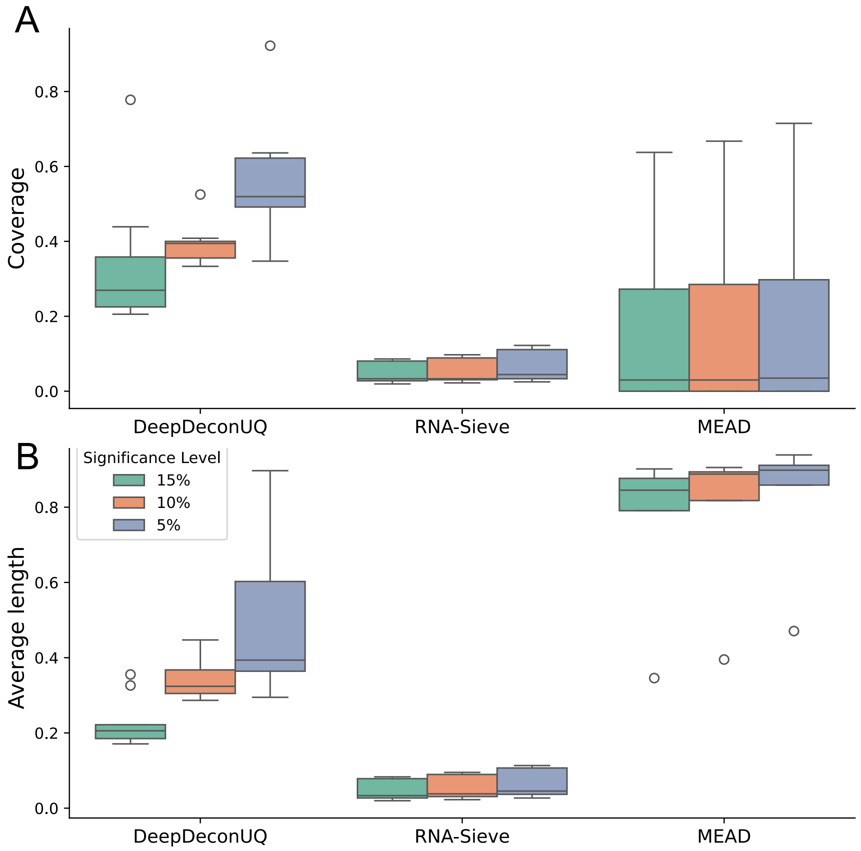

Fig D: **DeepDeconUQ outperforms other methods in predicting malignant cell type prediction interval on simulated Neuroblastoma bulk RNA-seq datasets.** Boxplots of coverage (A) and average prediction interval length (B) on simulated Neuroblastoma bulk RNA-seq datasets. Coverage is defined as the proportion of instances in which the true fraction of malignant cells falls within the prediction interval for the testing dataset. The average length represents the mean length of the prediction intervals across the testing datasets. Significance levels are indicated with different colors.

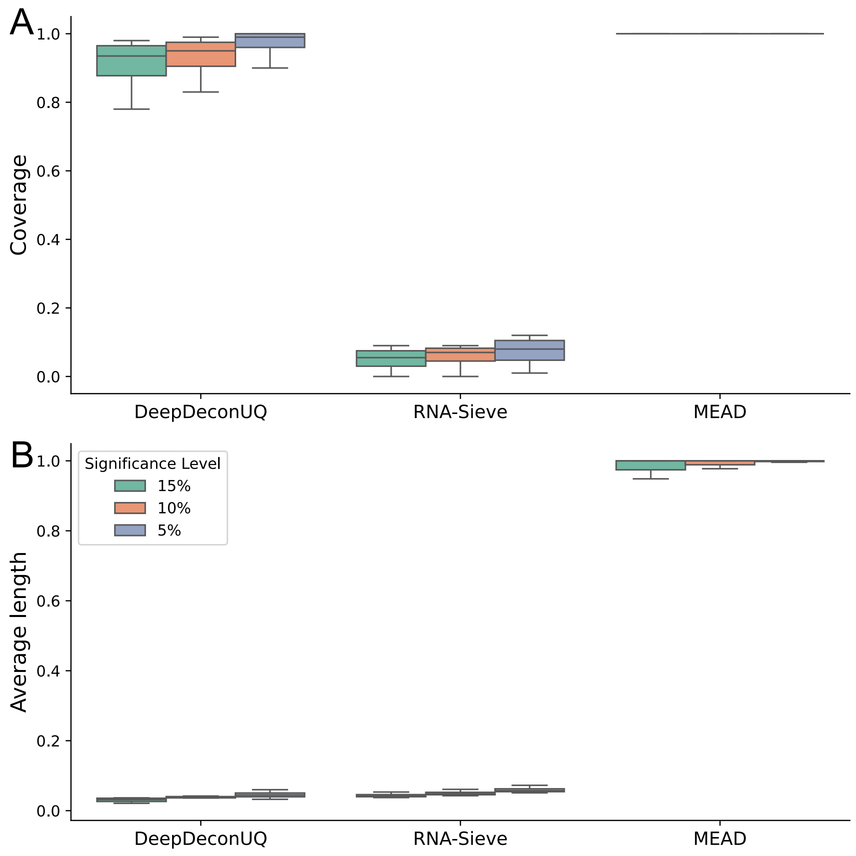

Fig E: **DeepDeconUQ outperforms other methods in predicting malignant cell type prediction interval on simulated epithelial bulk RNA-seq datasets.** Boxplots of coverage (A) and average prediction interval length (B) on four simulated epithelial bulk RNA-seq datasets. Coverage is defined as the proportion of instances in which the true fraction of malignant cells falls within the prediction interval for the testing dataset. The average length represents the mean length of the prediction intervals across the testing datasets. Each bar in the boxplot comprises 4 data points, each corresponding to one of 4 simulated epithelial datasets (except one dataset in MEAD that gives NA values and, therefore, doesn't show). Significance levels are indicated with different colors.

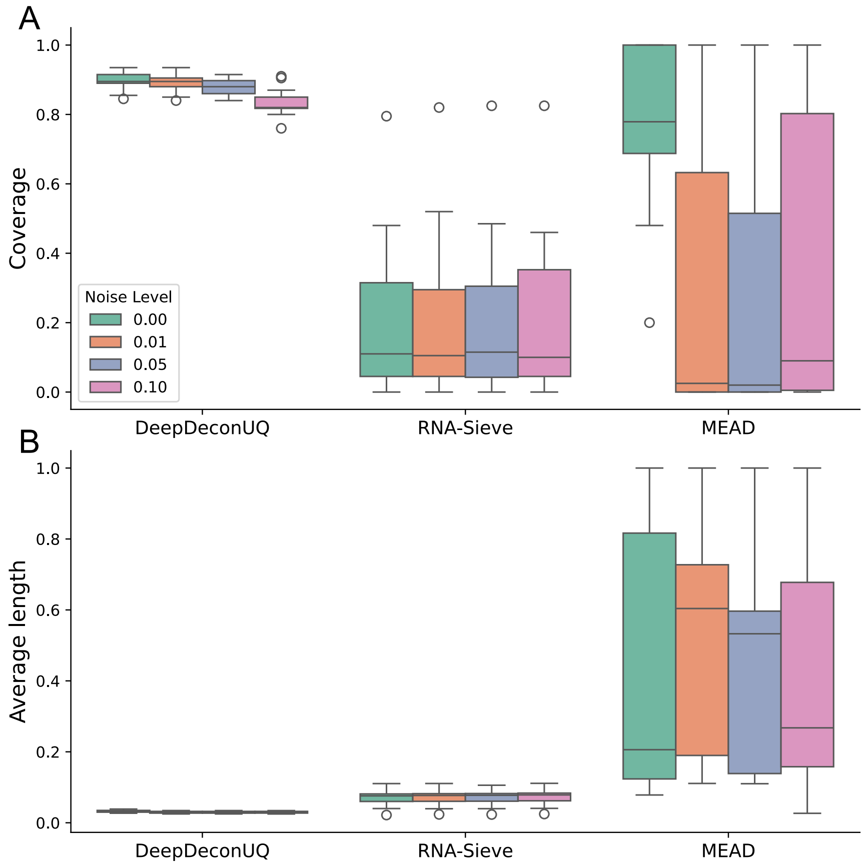

Fig F: **DeepDeconUQ is robust to gene expression perturbations.** Boxplots of coverage and average prediction interval length on 15 AML simulated bulk RNA-seq datasets under different noise levels. We added random noise generated from a Gaussian distribution with zero mean and variance that equals $\lambda(\lambda = 0.01, 0.05, 0.1)$ times the gene expression level for each gene in each sample. Each bar contains a total of 15 points, representing 15 separate AML datasets. The color represents different levels of noise level λ. Significance level $\alpha = 0.15$.

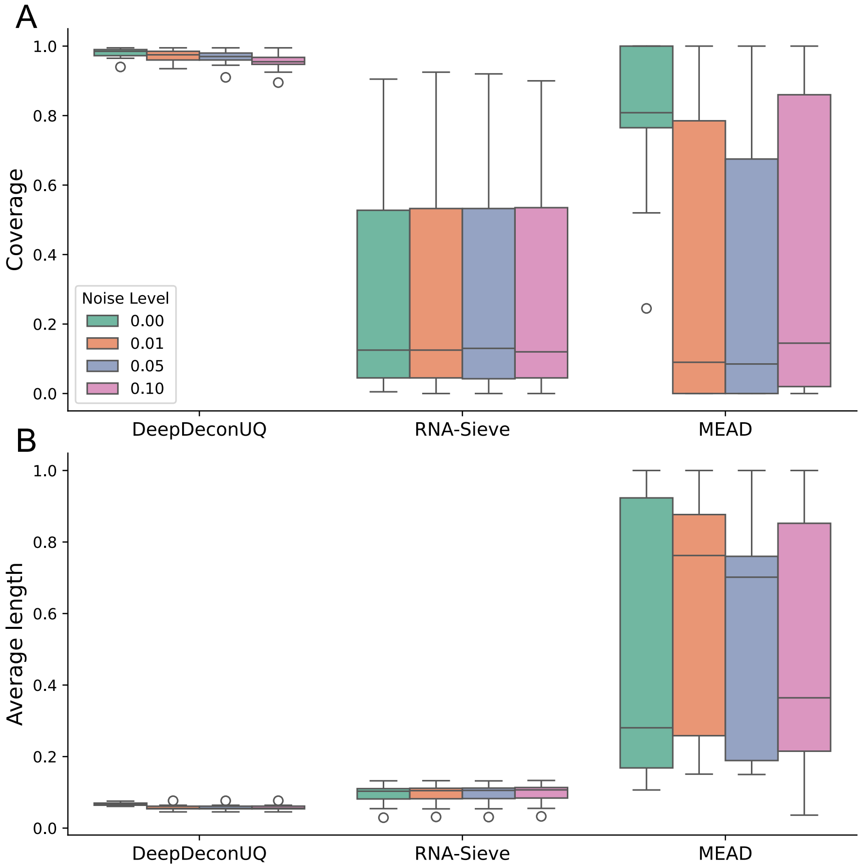

Fig G: **DeepDeconUQ is robust to gene expression perturbations.** Boxplots of coverage and average prediction interval length on 15 AML simulated bulk RNA-seq datasets under different noise levels. We added random noise generated from a Gaussian distribution with zero mean and variance that equals $\lambda(\lambda = 0.01, 0.05, 0.1)$ times the gene expression level for each gene in each sample. Each bar contains a total of 15 points, representing 15 separate AML datasets. The color represents different levels of noise level λ. Significance level $\alpha = 0.05$.

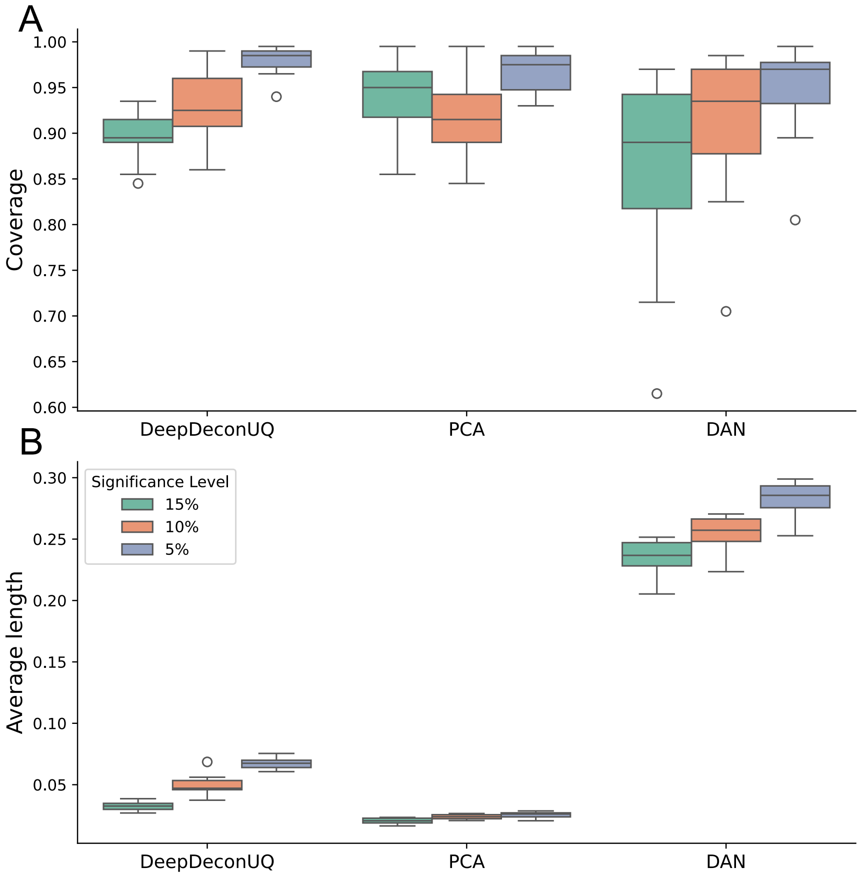

Fig H: **Comparison of DeepDeconUQ with different embedding methods.** Two embedding methods are used to compare with DeepDeconUQ. Principal Component Analysis (PCA) selects the top 100 principal components as neural network inputs. DAN makes use of transfer learning and generates a latent embedding layer for both training and testing datasets. Each point in the boxplot is an artificial bulk RNA-seq dataset.

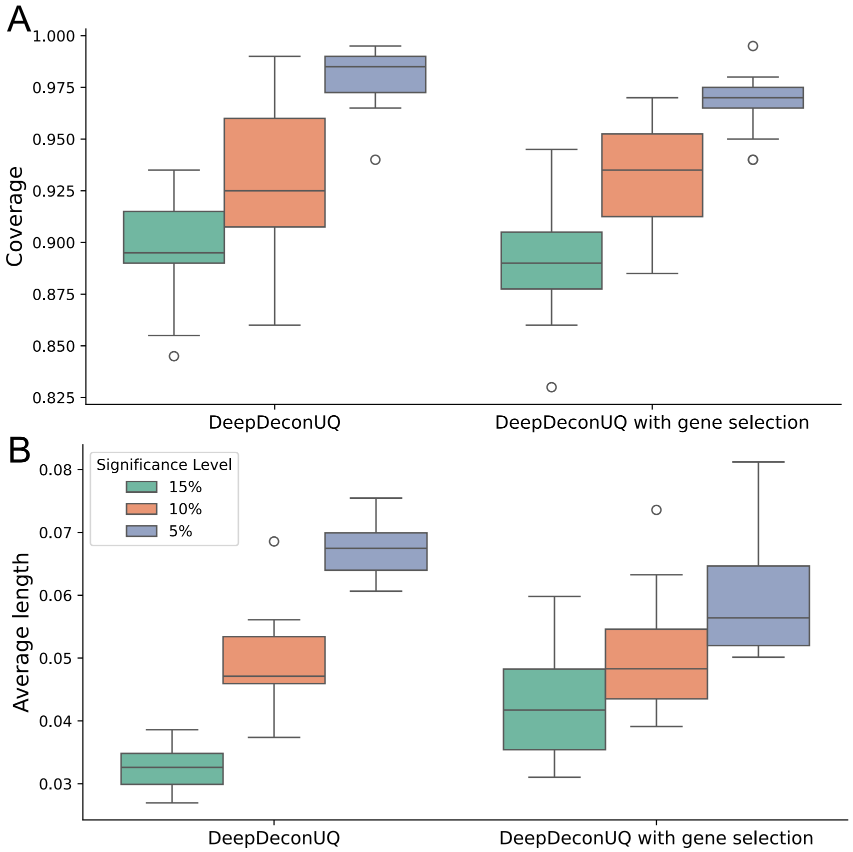

Fig I: **Comparison of DeepDeconUQ with/without gene selection on simulated AML datasets.** Each bar in the boxplot comprises 15 data points, each corresponding to one of 15 simulated epithelial datasets. Significance levels are indicated with different colors.

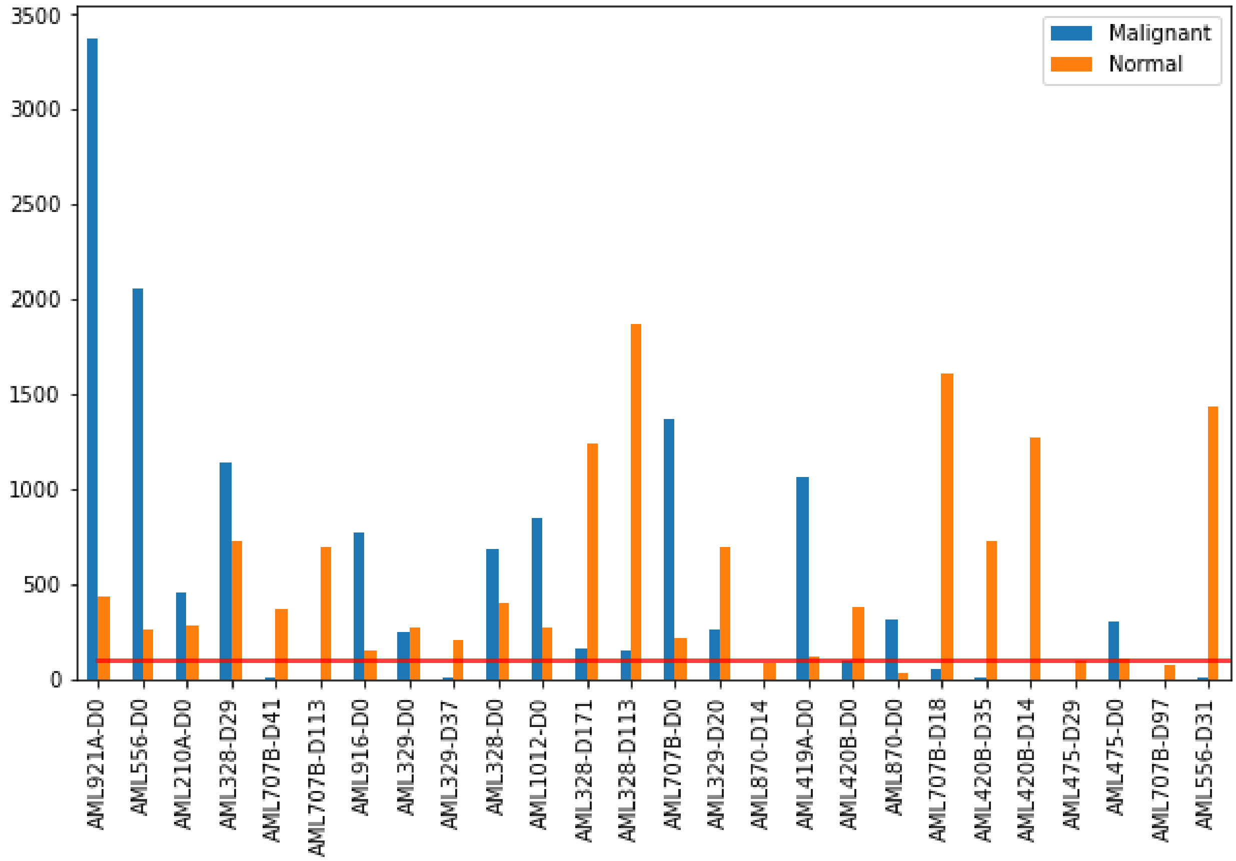

Fig J: **Barplots of the numbers of malignant and normal cells in each scRNA-seq subject.** Subjects with at least 100 malignant and 100 normal cells were selected for this study.

Table A: Performance of DeepDeconUQ with and without PCA embedding and with gene selection on real AML datasets.

**Methods**	**Dataset**	**15%**		**10%**		**5%**	
		**Coverage**	L_{avg}	**Coverage**	L_{avg}	**Coverage**	L_{avg}
DeepDeconUQ	primary	0.800	0.434	0.876	0.572	0.912	0.662
	recurrent	0.824	0.606	0.853	0.604	0.882	0.685
	beat	0.592	0.409	0.730	0.554	0.781	0.611
PCA embedding	primary	0.714	0.202	0.800	0.356	0.867	0.310
	recurrent	0.706	0.236	0.735	0.324	0.853	0.433
	beat	0.233	0.149	0.237	0.151	0.344	0.215
Gene Selection	primary	0.829	0.511	0.905	0.608	0.952	0.680
	recurrent	0.676	0.490	0.794	0.641	0.853	0.678
	beat	0.537	0.460	0.619	0.465	0.648	0.482

Table B: Preprocessing criteria for each subject in AML and neuroblastoma datasets. The gene expression threshold means the maximum gene expression value of a cell. The gene number threshold means the maximum number of expressed genes. This is to avoid gene expressions that do not represent a single cell. The criteria are based on Scanpy (v. 1.7.2) functions 'filter_cells' and 'filter_genes'.

Subject	Gene expression threshold	Gene number threshold
AML328-D29	7000	2500
AML1012-D0	5000	1600
AML556-D0	10000	3000
AML328-D171	5000	2000
AML210A-D0	6000	2000
AML419A-D0	7000	2500
AML328-D0	5000	2000
AML707B-D0	6000	2000
AML916-D0	5000	2000
AML328-D113	6000	2000
AML329-D0	8000	2000
AML420B-D0	7000	2000
AML329-D20	7000	2200
AML921A-D0	8000	2500
AML475-D0	4800	1500

Table C: Real bulk AML RNA-seq datasets used in DeepDecon

Name	Number of samples	Sequencing platform	Normalization method	Source
primary	117	Affymetrix Gene ST Array [9]	FPKM	GDC Data Portal [10]
recurrent	38	Affymetrix Gene ST Array	FPKM	GDC Data Portal
BeatAML	300	SureSelect [11]	CPM	Tyner, et al. 2018 [12]
Pediatric Neuroblastoma	99	Illumina Hi-Seq 2000 [13]	RPKM	cBioPortal [14]
TCGA-HNSC	518	Illumina Hi-Seq 2000	RPKM	LinkedOmics [15]

References

[1] Huang J, Du Y, Stucky A, Kelly KR, Zhong JF, Sun F. DeepDecon accurately estimates cancer cell fractions in bulk RNA-seq data. Patterns. 2024;5(5):100969. doi:10.1016/j.patter.2024.100969.

[2] van Galen P, Hovestadt V, Wadsworth M, Hughes T, Griffin GK, Battaglia S, et al. Single-Cell RNA-seq Reveals AML Hierarchies Relevant to Disease Progression and Immunity. Cell. 2019;176(6):1265–1281.e24. doi:10.1016/j.cell.2019.01.031.

[3] Matsumoto M, Nishimura T. Mersenne Twister: A 623-Dimensionally Equidistributed Uniform Pseudo-Random Number Generator. ACM Trans Model Comput Simul. 1998;8(1):3–30. doi:10.1145/272991.272995.

[4] Long M, Zhu H, Wang J, Jordan MI. Deep transfer learning with joint adaptation networks. In: International conference on machine learning. PMLR; 2017. p. 2208–2217.

[5] Sathe A, Mason K, Grimes SM, Zhou Z, Lau BT, Bai X, et al. Colorectal cancer metastases in the liver establish immunosuppressive spatial networking between tumor-associated SPP1+ macrophages and fibroblasts. Clinical Cancer Research. 2023;29(1):244–260. doi:10.1158/1078-0432.CCR-22-2041.

[6] Finak G, McDavid A, Yajima M, Deng J, Gersuk V, Shalek AK, et al. MAST: a flexible statistical framework for assessing transcriptional changes and characterizing heterogeneity in single-cell RNA sequencing data. Genome Biology. 2015;16:1–13. doi:10.1186/s13059-015-0844-5.

[7] Xie D, Wang J. Robust Statistical Inference for Cell Type Deconvolution. arXiv preprint arXiv:220206420. 2022;doi:10.48550/arXiv.2202.06420.

[8] Erdmann-Pham DD, Fischer J, Hong J, Song YS. Likelihood-based deconvolution of bulk gene expression data using single-cell references. Genome Research. 2021;31(10):1794–1806. doi:10.1101/gr.272344.120.

[9] Array AGS. TARGET's Study of Acute Myeloid Leukemia.;. `https://www.cancer.gov/ccg/research/genome-sequencing/target/using-target-data/technology#aml`.

[10] Portal GD. TARGET's Study of Acute Myeloid Leukemia.;. `https://gdc.cancer.gov/content/target-aml-publication-summary`.

[11] 38Mb S. Functional Genomic Landscape of Acute Myeloid Leukemia.;. `https://www.ncbi.nlm.nih.gov/projects/gap/cgi-bin/study.cgi?study_id=phs001657.v1.p1`.

[12] Tyner JW, Tognon CE, Bottomly D, Wilmot B, Kurtz SE, Savage SL, et al. Functional genomic landscape of acute myeloid leukaemia. Nature. 2018;562(7728):526–531. doi:10.1038/s41586-018-0623-z.

[13] 2000 IHS. Protocols used for TARGET's study of Neuroblastoma (NBL);. `https://www.cancer.gov/ccg/research/genome-sequencing/target/using-target-data/technology`.

[14] cBioPortal Pediatric Neuroblastoma. Neuroblastoma dataset from cBioPortal;. `https://www.cbioportal.org/study/summary?id=nbl_target_2018_pub`.

[15] Vasaikar SV, Straub P, Wang J, Zhang B. LinkedOmics: analyzing multi-omics data within and across 32 cancer types. Nucleic Acids Research. 2018;46(D1):D956–D963. doi:10.1093/nar/gkx1090.
